# A Single‐Year Cosmic Ray Event at 5410 BCE Registered in ^14^C of Tree Rings

**DOI:** 10.1029/2021GL093419

**Published:** 2021-06-09

**Authors:** F. Miyake, I. P. Panyushkina, A. J. T. Jull, F. Adolphi, N. Brehm, S. Helama, K. Kanzawa, T. Moriya, R. Muscheler, K. Nicolussi, M. Oinonen, M. Salzer, M. Takeyama, F. Tokanai, L. Wacker

**Affiliations:** ^1^ Institute for Space‐Earth Environmental Research Nagoya University Nagoya Japan; ^2^ Laboratory of Tree Ring Research University of Arizona Tucson AZ USA; ^3^ Department of Geosciences University of Arizona Tucson AZ USA; ^4^ Isotope Climatology and Environmental Research Centre Institute for Nuclear Research Debrecen Hungary; ^5^ Alfred Wegener Institute Helmholtz Centre for Polar and Marine Research Bremerhaven Germany; ^6^ Laboratory for Ion Beam Physics ETH Zürich Zürich Switzerland; ^7^ Natural Resources Institute Finland Rovaniemi Finland; ^8^ Faculty of Science Yamagata University Yamagata Japan; ^9^ Department of Geology Faculty of Science Lund University Lund Sweden; ^10^ Department of Geography Universität Innsbruck Innsbruck Austria; ^11^ Finnish Museum of Natural History University of Helsinki Helsinki Finland

**Keywords:** radiocarbon, tree rings, solar energetic particle, cosmogenic nuclide, solar activity

## Abstract

The annual ^14^C data in tree rings is an outstanding proxy for uncovering extreme solar energetic particle (SEP) events in the past. Signatures of extreme SEP events have been reported in 774/775 CE, 992/993 CE, and ∼660 BCE. Here, we report another rapid increase of ^14^C concentration in tree rings from California, Switzerland, and Finland around 5410 BCE. These ^14^C data series show a significant increase of ∼6‰ in 5411–5410 BCE. The signature of ^14^C variation is very similar to the confirmed three SEP events and points to an extreme short‐term flux of cosmic ray radiation into the atmosphere. The rapid ^14^C increase in 5411/5410 BCE rings occurred during a period of high solar activity and 60 years after a grand ^14^C excursion during 5481–5471 BCE. The similarity of our ^14^C data to previous events suggests that the origin of the 5410 BCE event is an extreme SEP event.

## Key Points

- Rapid ^14^C increase of ∼6‰ with significant level was found in tree rings from California, Switzerland, and Finland around 5410 BCE
- Five thousand, four hundred ten BCE event occurred during a period of high solar activity and 60 years after a grand ^14^C excursion of ∼20‰ from 5481 to 5471 BCE
- The similarity of this ^14^C signature to already known solar events suggests that the origin of this new 5410 BCE event is an extreme SEP

## 1 Introduction

The cosmic‐ray flux into the Earth atmosphere fluctuates over time due to various factors. Solar and geomagnetic modulation of galactic cosmic rays are dominating this process on timescales from a few‐days to millions of years, such as Forbush decreases (a shielding of galactic cosmic rays due to a coronal mass ejection), the 11‐year Schwabe cycle, multidecadal variation such as Grand Solar Minima, or millennial variations in Earth's magnetic field strength. In addition to these recurring factors, sporadic and intense solar energetic particle (SEP) events can also contribute to a sudden ejection of cosmic rays to the Earth. A large‐scale SEP event reaching Earth is known as a ground level enhancement (GLE), which can be detected as a large increase in the count rates of ground‐based neutron monitors. Since 1942, 72 GLEs have been recorded (1: GLE database). Prior to direct observations of cosmic rays, signatures of past extreme SEP events can be recorded by cosmogenic nuclides, in particular, ^14^C in tree rings, and ^10^Be and ^36^Cl in polar ice cores, all of which are produced in a particle cascade triggered by interactions of high energy cosmic rays with the constituents of Earth's lower atmosphere (Mekhaldi et al., 2015; Miyake, Usoskin, & Poluianov, 2019).

Conceivable SEP‐driven cosmic ray events reported so far in multiple studies are the 774/775 CE (Büntgen et al., 2018; Güttler et al., 2015; Jull et al., 2014; Mekhaldi et al., 2015; Miyake et al., 2012, 2015; Park et al., 2017; Rakowski et al., 2015; Scifo et al., 2019; Sigl et al., 2015; Usoskin et al., 2013; Uusitalo et al., 2018), 992/993 CE (Büntgen et al., 2018; Fogtmann‐Schulz et al., 2017; Miyake et al., 2013, 2014; Miyake, Horiuchi, et al., 2019; Mekhaldi et al., 2015; Rakowski et al., 2018; Scifo et al., 2019), and ∼660 BCE events (O'Hare et al., 2019; Park et al., 2017; Sakurai et al., 2020). All these events have been reported as rapid increases of cosmogenic nuclides concentrations (^10^Be, ^14^C, and ^36^Cl) within a year. The magnitude of the SEP events that might cause these signals in the proxy records is estimated to be an order of magnitude higher than the largest instrumentally measured GLE (Mekhaldi et al., 2015; O'Hare et al., 2019; Usoskin et al., 2013). It is important to illuminate the occurrence rate and intensity of similar cosmic ray events not only for solar physics research but also for the forecasting of space weather. In previous attempts to search for similar events, the origins of other ^14^C excursions in tree rings have not been clearly detected except for the above‐mentioned three events (Miyake, Masuda, et al., 2017). Wang et al. (2017) reported a fourth SEP event that might have occurred in 3372/3371 BCE using tree rings of a subfossil specimen of Chinese wingnut buried in a riverbank. The ring cross‐dating and the date of this event were questioned by Jull et al. (2021). Their study of ^14^C variations in absolutely dated rings of California bristlecone pine and German oak could not reproduce the event (Jull et al., 2021). Therefore, the date, if not the presence, of the 3372/3371 BCE event should be noted as controversial up to now. Also, rapid ^14^C increases have been reported in 1528 BCE (Pearson et al., 2020), ∼1055 CE (Brehm et al., 2021; Eastoe et al., 2019; Terrasi et al., 2020), and 1279 CE (Brehm et al., 2021). However, the ^14^C increases in 1528 BCE and 1279 CE were each reported by only one tree and location (Brehm et al., 2021; Pearson et al., 2020). The ^14^C increase around 1055 CE was suggested as a different ^14^C variation from the SEP‐driven events and having a possible link with the supernova SN 1054 (Terrasi et al., 2020). Therefore, detailed verifications using multiple trees and multi‐nuclides will be needed to confirm the occurrence and the origin of these newly registered events.

For a further search of cosmic ray events, the use of tree‐ring ^14^C is more beneficial than other cosmogenic nuclides proxies, since ^14^C concentration in a tree specimen can be regarded as representative of the troposphere of that hemisphere. This allows us to rely on samples from only one tree specimen in the event search. Another benefit of using the tree‐ring proxy is the precise and absolute dating of tree‐ring samples back to 12,310 cal BP with the methods of dendrochronology called cross‐dating that assigns the calendar years to tree rings (Fritts, 1976; Stokes & Smiley, 1968). The signature of ^14^C content in tree‐ring series of the mentioned above SEP‐driven events involves a sharp single‐year jump of Δ^14^C following a 2–3 year gradual increase (or a plateau) and a subsequent decrease of Δ^14^C to the normal values. This particular signature of tree‐ring ^14^C is easily recognized as an abnormal change in the ^14^C production rate. In order to detect more events, we analyzed the annual variability of ^14^C concentration in three independent tree‐ring records from California, Switzerland, and Finland for the period from 5421 to 5371 BCE. This period follows a large ^14^C excursion around 5480 BCE found earlier (Miyake, Jull, et al., 2017). So far, this excursion is considered to be the largest one in the Holocene.

## 2 Materials and Methods

### 2.1 Tree‐Ring Samples

We used three tree specimens from three geographical locations far distant from each other, for which the ^14^C annual records were independently produced and compared. One of them is a Pinus longaeva specimen 1971#059 from the Methuselah Walk site in the White Mountains of California, United States (37.3794°N, 118.1654°W, 3,094 a.s.l., Figure S1). The remnant wood specimen was collected in 1971 and stored at Laboratory of Tree‐Ring Research (LTRR) archive of the University of Arizona. This is the same specimen used for the 5480 BCE excursion in Miyake, Jull, et al. (2017). Calendar dates of bristlecone pine rings were established with cross‐dating of tree‐ring width measurements matching the 1971#059 series against the Methuselah Walk master chronology (LaMarche & Harlan, 1973). The Methuselah Walk chronology is one the best cross‐dated records of bristlecone pine that originally was built in the early 1970s and updated twice in the 1980s and then 2010s (Ferguson & Graybill, 1983; LaMarche & Harlan, 1973; Salzer et al., 2019). The second set of the tree rings originates from a Pinus sylvestris specimen of Finnish Lapland (69.26°N, 27.40°E, 199 a.s.l., Figure S1). The specimen comes from a subfossil tree trunk preserved in anoxic conditions of lacustrine sediments and excavated during the large‐scale field campaign to build a subfossil pine network for the region (Helama et al., 2019). Tree‐ring series of the specimen spans between 5562 and 5331 BCE interval and is cross‐dated against the Finnish super long master chronology (Helama et al., 2008, 2019). The last tree‐ring specimen comes from a Larix decidua Mill. trunk found on the forefield of the Unteraar glacier, Switzerland (46°34′ N, 8°15′ E, 1,930 m a.s.l., Figure S1). The log ua1307 was buried in sediments and these were at least temporarily covered by the glacier. The log was sampled in 2013 during field activities for collecting tree‐ring material on Holocene glacier variability. The ring width series of the specimen dates to the period 5459–5368 BCE and cross‐dated against the Eastern Alpine Conifer Chronology as the reference chronology (Nicolussi et al., 2009). Here, we use Gregorian calendar year BCE (not including year zero between BCE and CE) throughout the text for the convenience of readers. However, we use astronomical year (minus age: including year zero delineated Common Era from the time before) for the graphs.

### 2.2 Pretreatments and ^14^C Measurements

Each annual ring was separated for the period 5420–5396 BCE (California), 5421–5371 BCE (Switzerland), and 5420–5400 BCE (Finland). Since continuous ^14^C data of the California tree for the period of 5420–5412 BCE had already been taken (Miyake, Jull, et al., 2017), we newly prepared a set of samples for the period of 5411–5396 BCE. Each annual ring of the California and Finnish specimens was converted to cellulose samples by the method described in Miyake, Jull, et al. (2017). The ^14^C concentration in the California specimen was measured using accelerator mass spectrometry at ETH Zürich and Yamagata University (YU‐AMS, Tokanai et al., 2013), and that in the Finnish specimen was measured once or in some cases, twice at YU‐AMS. The Switzerland specimen was treated to cellulose by the BABAB method (Němec et al., 2010) and ^14^C concentrations were measured 1–3 times at ETH Zürich (Sookdeo et al., 2020). The Δ^14^C datasets are provided in supporting information (Table S2).

## 3 Results and Discussions

### 3.1 Significance of the Event

**Figure 1:**
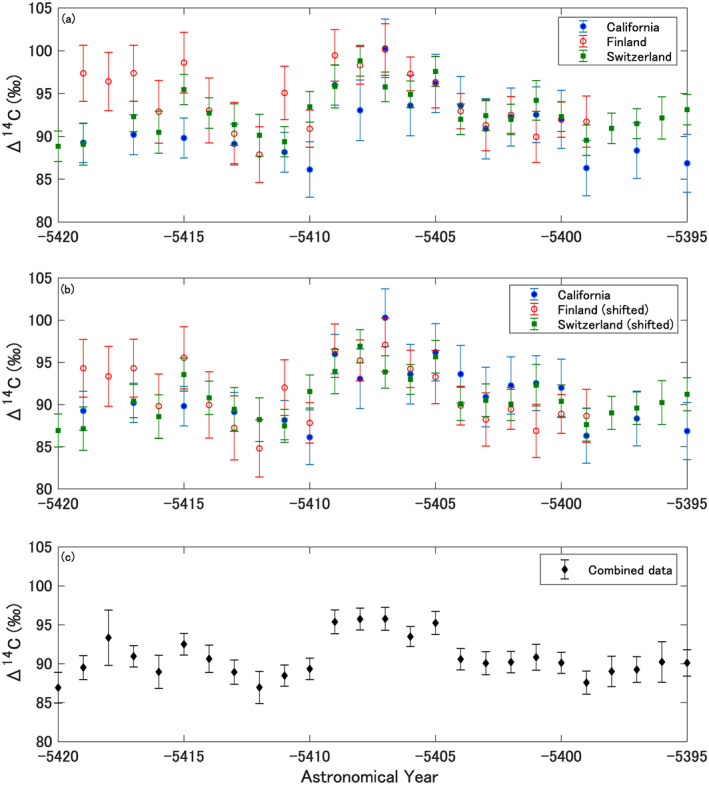
(a) Comparison of Δ^14^C values between the California bristlecone pine, Switzerland larch, and Finland Scots pine series. (b) The same as Figure 1a, but the Switzerland and Finnish series are shifted as minus 1.9‰ and 3.1‰ in the vertical axis direction. (c) Combined Δ^14^C values (weighted averages) of the three series in Figure 1b.

Figure 1a shows measured ^14^C annual data for the tree‐ring series from California, Switzerland, and Finland. Since the ages measured multiple times for each tree series are in a good agreement (no data is rejected at the 95% confidence level by a chi‐square test) we combined them via weighted averaging. It is found in our data that the means of Δ^14^C values become higher with latitude, which has been suggested in the previous literature (Büntgen et al., 2018). The offsets are 1.9 ± 0.8‰ and 3.1 ± 1.0‰ on average between the series from California and Switzerland, and California and Finland, respectively (Figures S2 and S3). A T‐test estimates that the offset between the series from California and Finland is significant (*p* = 0.002). Büntgen et al. (2018) reported an approximately 3‰ and 2‰ offsets observed between the atmospheric radiocarbon zones NH0 and NH2, and NH0 and NH1, respectively (NH0, NH1, and NH2 correspond to the geographical locations of our Finnish, Switzerland, and Californian specimens), and the range of our offsets has a close similarity to the reported offsets. Note that these radiocarbon zones were defined for the ^14^C data after the periods of the atmospheric bomb peak (Hua et al., 2013), and the NH0 zone was added to see the effect of the high‐latitude region by Büntgen et al. (2018). Therefore, we consider the measured offset as the fingerprint of increasing ^14^C concentrations with latitude across the hemisphere. Our study found a significant annual increase of ^14^C concentrations from 5411 to 5410 BCE in the California and Finland tree‐ring records. The increment between these two years is 9.9‰ in California series and 8.6‰ in the Finnish one, which corresponds to 3.5 and 3.3 times of the measurement error, respectively. Although the Swiss series does not show a significant increase from 5411 to 5410 BCE, a ∼10‰ increase is observed from 5412 to 5409 BCE. This Δ^14^C variation matches the two other series well considering the measurement uncertainties. For further analysis, we subtracted the offsets between the data series so that the Swiss and Finnish series match the Δ^14^C from California. Figures 1a and 1b compares all three tree‐ring series. After subtraction of the offset, the remaining scatter between the datasets is consistent with the measurement uncertainties and no data rejected at 95% confidence by a chi‐square test. The combined series (Figure 1c) shows a 6.0‰ increase in Δ^14^C from 5411 to 5410 BCE, which is statistically significant and 4.2 times larger than the measurement uncertainties. After the rapid increase of 5411/5410 BCE, the high Δ^14^C values are sustained for 3 years with the peak occurring in 5408 BCE and then gradually decreasing. Hereafter, we will refer to this signature in the Δ^14^C variation as the 5410 BCE event.

### 3.2 Comparison With Other Annual Cosmic Ray Events

**Figure 2:**
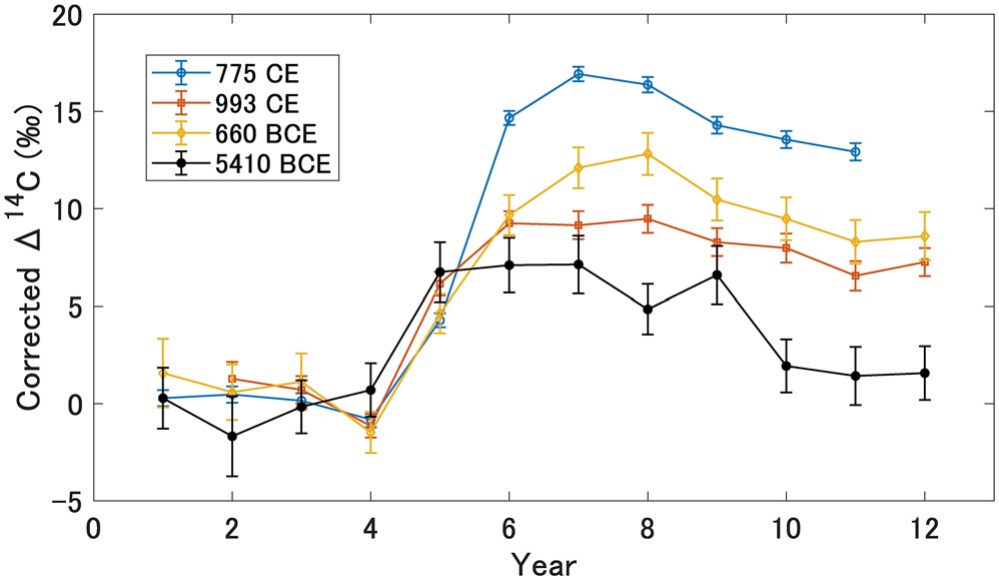
Comparison of the known annual cosmic ray events. The 774/775 CE event: open circles (Büntgen et al., 2018: mean of 26 sites from northern hemisphere), the 992/993 CE event: open squares (Büntgen et al., 2018: mean of seven sites from northern hemisphere), the ∼660 BCE event: open diamonds (Sakurai et al., 2020), and the 5410 BCE event: solid circles (this study). The horizontal line represents time in the unit of year, and the significant increases occur in year no.5. The vertical lines are shifted so that each baseline has an average of zero. The baselines are defined as the weighted average of the data before the significant increases (data of no. 1–4 or no. 2–4).

The ^14^C increase during the 5410 BCE event is very similar to the structure of annual cosmic ray events detected so far, that is, a single‐year sharp Δ^14^C increase followed by a plateau (Figure 2). To compare the ^14^C variation of the 5410 BCE event with other known ^14^C excursions, we used the high‐resolution datasets of Δ^14^C values from Büntgen et al. (2018) for the 774/775 CE and 992/993 CE events, and from Sakurai et al. (2020) for the 660 BCE event. Since the data of Sakurai et al. (2020) were taken with intra‐seasonal resolution (earlywood and latewood separate analysis), we averaged them into one annual series just as shown in Figure 2 of Sakurai et al. (2020). The comparison indicates a significant increase of Δ^14^C (>3 times larger than measurement uncertainties) during the cosmic ray events of 774 CE, 993 CE, 664 BCE, and 5410 BCE. Note that the baseline of each event (zero of *Y* axis in Figure 2) is the weighted average of four data points (except the 993 CE event with three data points) preceding the significant shift in Δ^14^C. The comparison suggests that the magnitude of the 5410 BCE event is lower than in the previously reported cosmic ray events. The maximum ^14^C increase at 5408 BCE is very close to the 992/993 CE event, though the errors of the 5410 BCE event are larger (Table S1). The similarity of the event signatures implies that a short‐term increase in the cosmic‐ray flux occurred in 5411/5410 BCE. It has been discussed that an extreme SEP may not be the only cause of these rapid increases in ^14^C production. Galactic phenomena resulting from gamma rays (supernova explosion and gamma ray burst in Hambaryan & Neuhäuser, 2013; Pavlov et al., 2013) are possible explanations. At this stage where only ^14^C data from the tree rings are available, we cannot exclude these other phenomena as the cause of the 5410 BCE event. We note that the signal strength seems not to be higher at the higher latitude as it was observed for 774/775 CE event (Uusitalo et al., 2018) and shown globally (Büntgen et al., 2018). In order to determine the specific origin, the enhancement‐ratio of other cosmogenic nuclides (^14^C, ^10^Be, and ^36^Cl) in ice cores and the meridional gradient of cosmogenic nuclides need to be investigated (Büntgen et al., 2018; Mekhaldi et al., 2015; Miyake, Usoskin, & Poluianov, 2019; Uusitalo et al., 2018).

### 3.3 Solar Activity of the Sixth Millennium BCE: Forerunner of the Mid‐Holocene

**Figure 3:**
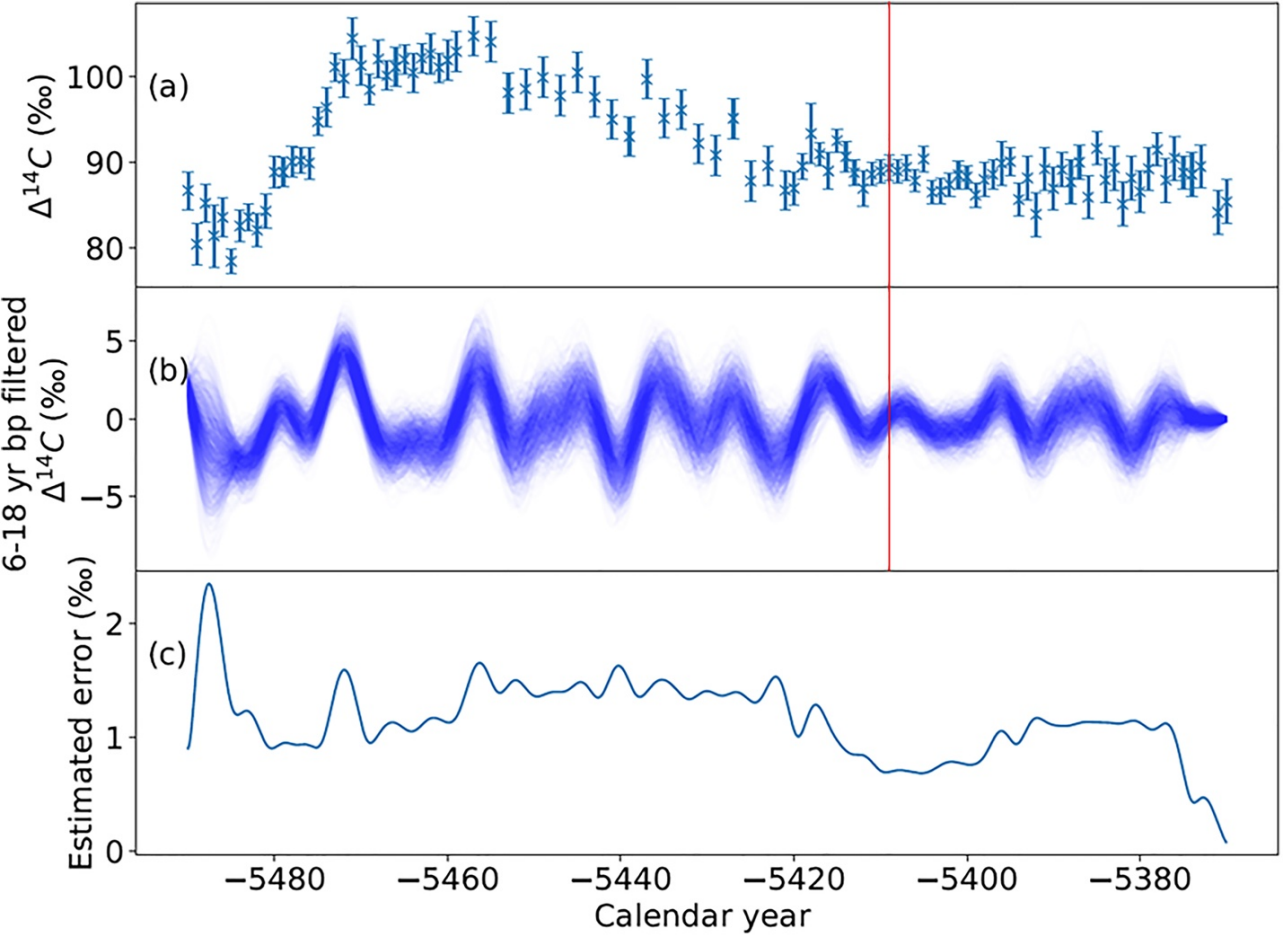
(a) Δ^14^C data set after the 5410 BCE event was subtracted by a box‐model simulation (see text). (b) band‐pass filtered series of 1,000 generated data series with 6–25 years bandwidth of the Δ^14^C data in Figure 3a from 5491–5371 BCE. The red line indicates 5410 BCE. (c) Estimated errors calculated from the standard deviation of 1,000 randomly generated data series.

Figure S3 shows the data of this study (Figure 1c series) and the data published in Miyake, Jull, et al. (2017) dated to sixth millennium BCE. During the period of 5482–5472 BCE, a large excursion in ^14^C concentrations is observed, the cause of which has not yet been determined (Miyake, Jull, et al., 2017), but, due to the similarity with other excursions, is likely to be a grand solar minimum. After the large excursion, the data show a gradual decrease for about 60 years before the 5410 BCE event reported here appears. Since the ^14^C variation during the gradual decrease is very similar to the end of the Maunder minimum (well‐defined and best studied grand solar minimum) in 1640–1720 CE, the gradual decrease period might represent an increase of solar activity, as solar magnetic activity gradually builds up. Such a characteristic is also common to the 775 CE and 660 BCE events, that is, the events that have occurred during increasing solar activity levels, that is decades after the end of Grand Solar Minima. Although further verification is needed to specify the origin of the 5410 BCE event, it is noteworthy that the 5410 BCE event occurred during a period of high solar activity. During the gradual decay‐phase of the event, there are no rapid changes in Δ^14^C, only rather small regular fluctuations which might be caused by the Schwabe cycle. Since other studies successfully applied a bandpass filter to detect the Schwabe cycle in ^14^C ring records (e.g., Güttler et al., 2013; Scifo et al., 2019; Vasil'ev et al., 2019), we used a similar approach (6–25 year bandpass analysis) for the period of 5491 BCE to 5371 BCE using data from Miyake, Jull, et al. (2017) and this study (Figure 3a). First, to subtract the effect of the 5410 BCE event, we fitted the Δ^14^C data from 5414 to 5396 BCE by using a 22‐box carbon cycle model (Büntgen et al., 2018) assuming a pulsed ^14^C production with 4.6 × 10^26^ atoms occurred in 5410 BCE (Figure S4). Figure 3a shows a subtracted data set. We applied 6–25 years bandpass analyses for 1,000 generated data series using the ^14^C data and their errors of Figure 3a (Figure 3b) to detect any possible cyclicity around 11 years in our data set. Figure 3c shows the estimated errors of Figure 3b, which is the standard deviations of 1,000 randomly generated data series. The result shows a periodic variation around the Schwabe cycle length over the entire period, which are strongly influenced by the measurement uncertainties for individual tree‐rings of about 2‰. Brehm et al. (2021) observed that the measurement noise primarily leads to an over‐estimation of amplitudes after bandpass filtering. We therefore generated 5,000 synthetic, random datasets around a sine‐wave of a certain amplitude and cycle length with the same sampling resolution based on the uncertainties of the measurement series to estimate their influence on amplitude and a cycle length. The random datasets produced for different cycle lengths and amplitudes were likewise 6–25 year bandpass filtered before the histograms of peak amplitudes and the peak‐to‐peak distances were generated and compared to the ones obtained for measured data set. The comparison is shown in a probability map in Figure S6. A 11‐year periodic variation with the amplitude modulation of 0.7–1.6‰ (1‐sigma range) explains the data set well, consistent within error with reconstructions for the last millennium (Brehm et al., 2021) but is significantly less than the 6‰ increase observed for the 5410 BCE event. The Schwabe cycle manifested in the Δ^14^C data is expected to lag the actual solar cycle by 2–3 years because of the large and exchanging carbon reservoirs in the carbon cycle (e.g., Siegenthaler et al., 1980). For example, a 3 ± 1 year lag was observed in the recent verification of sunspot fluctuations around the 1860s using tree‐ring ^14^C data (Scifo et al., 2019). The 5410 BCE event occurred during a solar maximum which, considering this 3‐year lag, is likewise consistent with the results of the 774/775 and 992/993 CE SEP events (Scifo et al., 2019). Additional cosmogenic isotope data are needed to confirm the cause of the 5410 BCE event, however, its association with increasing solar activity indicates that another extreme SEP event is a plausible explanation of its origin.

## 4 Conclusions

We found a significant ^14^C increase in 5411/5410 BCE from the annual ^14^C analysis that has been replicated in three tree‐ring records from California, Switzerland and Finland. The ^14^C variation is similar to the SEP‐driven ^14^C events in the 774/775 CE, 992/993 CE, and ∼660 BCE. The magnitude of the 5410 BCE event is slightly below the 992/993 CE event. Although the cause of the event signal has just been hypothesized, it is consistent with the origin of the extreme SEP event based on the similarity to the other confirmed SEP events. It is likely that the 5410 BCE event occurred during a period of increasing long‐term solar activity as well as during the maximum of a Schwabe cycle. If this event originates from a SEP, this finding will contribute to better estimate the frequency and intensity of extreme SEP events and their relationship to the solar activity.

## Supporting information

Supporting Information S1

Table S1

## Data Availability

Datasets for this research are available at the supporting information (Table S2), and https://doi.org/10.34515/DATA.C14-00001.
