# Supplementary material for: A Single‐Year Cosmic Ray Event at 5410 BCE Registered in 14C of Tree Rings
Source: Geophys Res Lett. 2021 Jun 9;48(11):e2021GL093419. doi: 10.1029/2021GL093419 (PMC8365682; doi:10.1029/2021GL093419)
Supplement: Supplementary file 1 — Supporting Information S1 [file GRL-48-e2021GL093419-s001.docx]

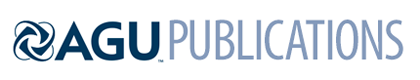


*[Geophysical Research Letters]*

Supporting Information for

**[A single-year cosmic ray event of 5410 BCE registered in ^14^C of tree rings]**

[F. Miyake^1^, I. P. Panyushkina^2^, A. J. T. Jull^3,4^, F. Adolphi^5^, N. Brehm^6^, S. Helama^7^, K. Kanzawa^1^, T. Moriya^8^, R. Muscheler^9^, K. Nicolussi^10^, M. Oinonen^11^, M. Salzer^2^, M. Takeyama^8^, F. Tokanai^8^, L. Wacker^6^

]

[^1^Institute for Space-Earth Environmental Research, Nagoya University, Furo-cho, Chikusa-ku, Nagoya, 464-8601, Japan, ^2^ Laboratory of Tree Ring Research, University of Arizona, Tucson, AZ 85721, ^3^ Department of Geosciences, University of Arizona, Tucson, Arizona USA, ^4^ Isotope Climatology and Environmental Research Centre, Institute for Nuclear Research, Debrecen, Hungary, ^5^ Alfred Wegener Institute, Helmholtz Centre for Polar and Marine Research, Bremerhaven, Germany, ^6^ Laboratory for Ion Beam Physics, ETH Zürich, CH-8093 Zürich, Switzerland, ^7^ Natural Resources Institute Finland, Ounasjoentie 6, 96200 Rovaniemi, Finland, ^8^ Faculty of Science, Yamagata University, 1-4-12 Kojirakawa-machi, Yamagata, 990-8560, Japan, ^9^ Department of Geology, Faculty of Science, Lund University, Sölvegatan 12, 223 62 Lund, Sweden, ^10^ Department of Geography, Universität Innsbruck, Innrain 52, 6020 Innsbruck, Austria, ^11^ Finnish Museum of Natural History, 00014 University of Helsinki, Finland]

**Contents of this file**

Figures S1-S5

Table S1

**Additional Supporting Information (Files uploaded separately)**

Table S2 (Datasets)


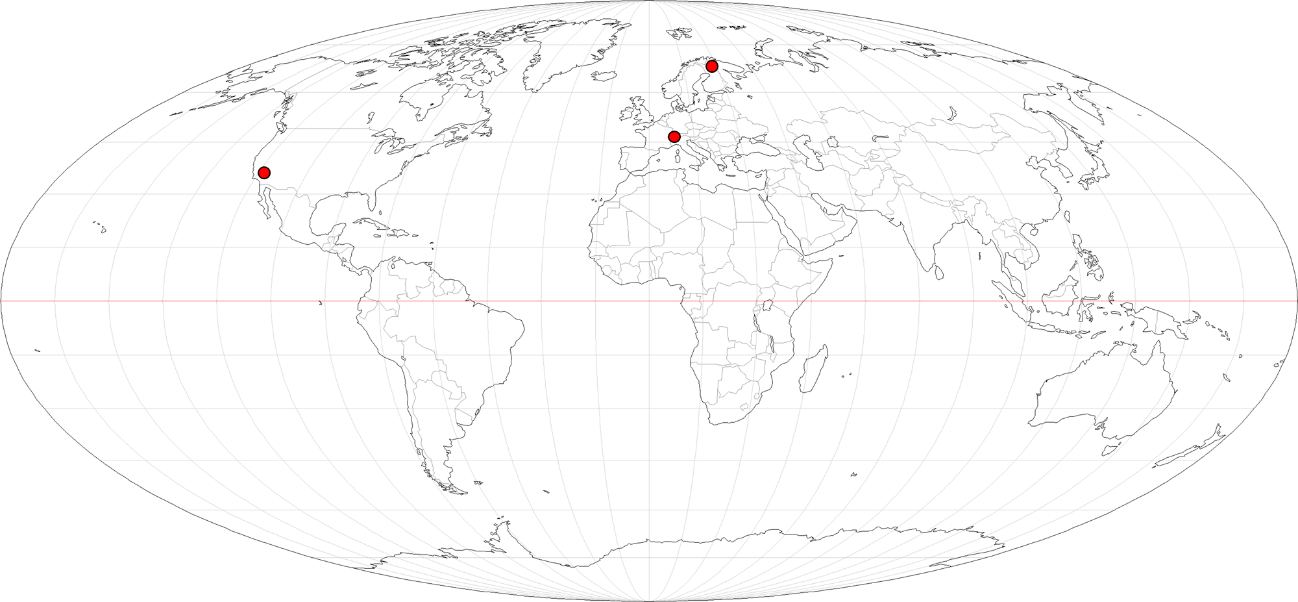


Figure S1. Sample locations. We used a bristlecone pine specimen from California, U.S. (37.3794°N, 118.1654°W, 3094 m a.s.l.), larch specimen from Switzerland (46.57°N, 8.25°E, 1930 m a.s.l.), and a scots pine specimen from Finnish Lapland (69.26°N, 27.40°E, 199 m a.s.l.).

Figure S2. Difference of 14C concentrations between the Finland and California tree specimens. The Finland series is on average (3.1±1.0)‰ higher in 14C than the California series.

Figure S3. Difference of 14C concentrations between the Switzerland and California tree specimens. The Switzerland series is on average (1.9±0.8)‰ higher in 14C than the California series.

Figure S4. Δ^14^C values of Miyake et al. (2017b) which used the California tree specimen, and this study (combined data after subtracted the offsets to match the California series).


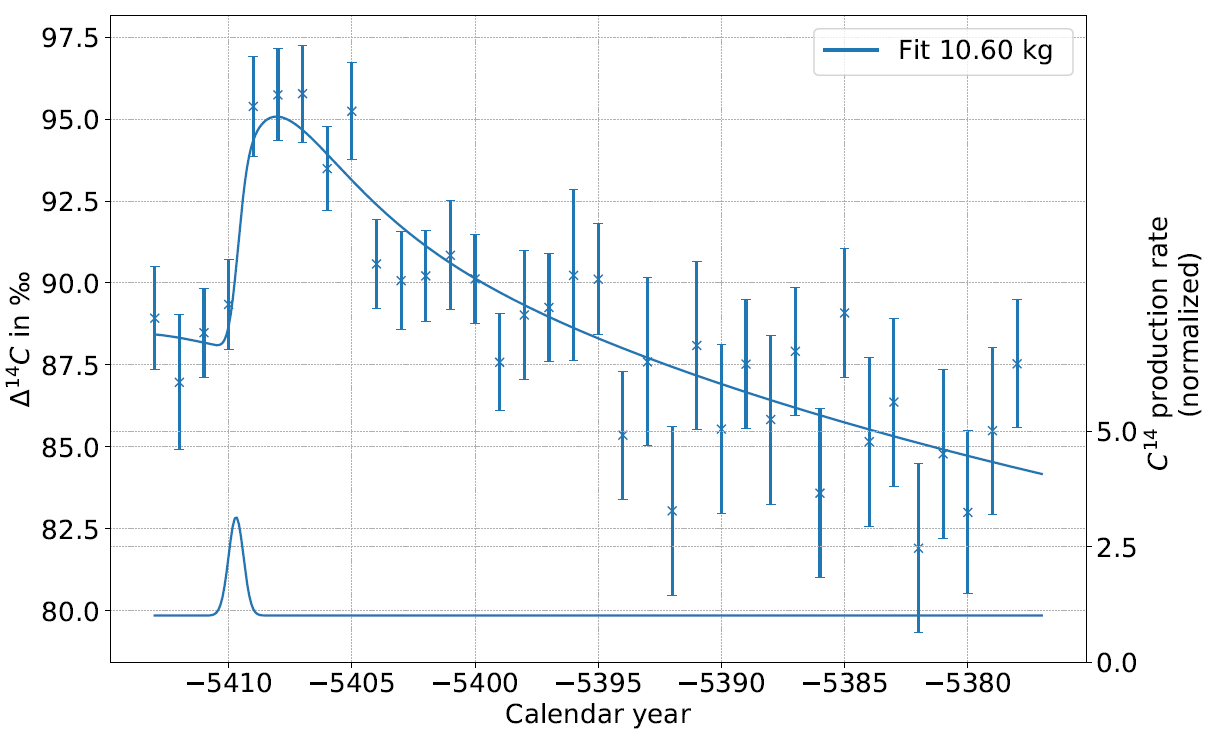


Figure S5. Fitting of the 5410 BCE event by a carbon cycle box model simulation with an additional production input of 10.60 kg of ^14^C (Brehm et al. 2020).


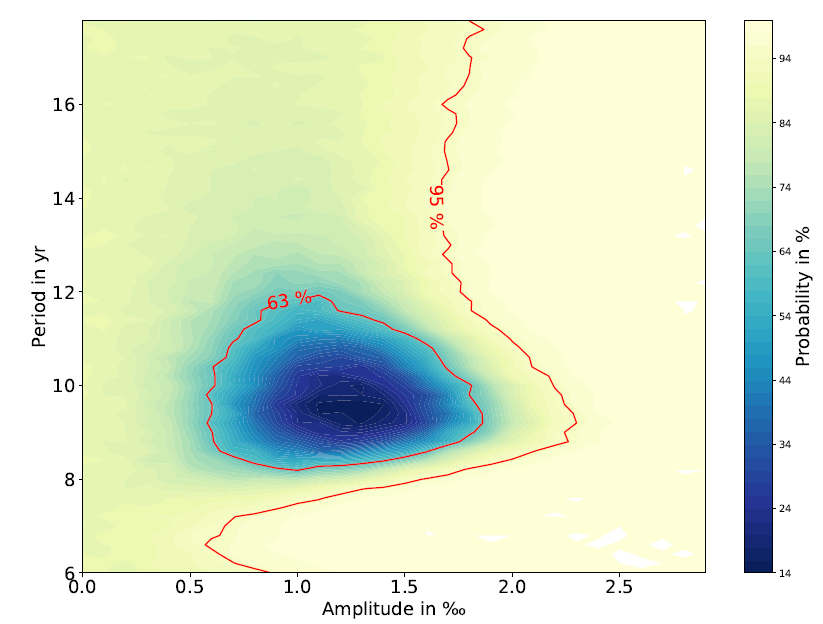


Figure S6. Probability map of the band pass analysis against the amplitudes (x-axis) and periods (y-axis). 1-and 2-sigma ranges are indicated by the red lines

Table S1. List of single-year cosmic ray events. These values are obtained from the data of figure 2 (see text. 775 CE event and 993 CE event: Büntgen et al. 2018, ~660 BCE event: Sakurai et al. 2020). Here, errors of baseline are standard deviation.

| Event | Year of significant  increase | Maximum increment  from baseline (‰) | Year of maximum increment |
| --- | --- | --- | --- |
| 775 CE | 774 CE | 16.9 ± 0.4 | 776 CE |
| 993 CE | 993 CE | 9.5 ± 1.5 | 996 CE |
| ~660 BCE | 664 BCE | 12.8 ± 1.7 | 661 BCE |
| 5410 BCE | 5410 BCE | 7.1 ± 1.8 | 5408 BCE |
